# Role of FEF25–75 in characterizing outpatients with asthma in clinical practice 

**DOI:** 10.5414/ALX02453E

**Published:** 2024-01-12

**Authors:** Giorgio Ciprandi, Irene Schiavetti

**Affiliations:** 1 Allergy Clinic, Department of Outpatients, Casa di Cura Villa Montallegro, and; 2 Health Science Department, University of Genoa, Genoa, Italy

**Keywords:** asthma, FEF25, 75, FEV1, bronchial obstruction, asthma control, clinical presentation, clinical practice, real-life, spirometry, physical examination

## Abstract

Background: Asthma is characterized by variable airflow limitation. FEF_25–75_ has been proposed as a reliable marker for bronchial obstruction, especially when FEV_1_ and FEV_1_/FVC are normal. Objectives: To investigate the role of FEF_25–75_ in patients with asthma seen in clinical settings. Materials and methods: The cross-sectional study included 439 (181 females and 255 males; mean age 39 years) outpatients with asthma who consecutively visited an allergy clinic for a routine assessment. History, physical examination, asthma control, and spirometry were evaluated. Results: FEF_25–75_ was impaired (< 65% of predicted) in 136 (31%) outpatients. Considering only subjects with normal FEV_1_ and FEV_1_/FVC, FEF_25–75_ was impaired in 71 (19.6%) subjects. In this subset, impaired FEF_25–75_ was associated with low FEV_1_ and FEV_1_/FVC values (OR 0.91 and 0.85, respectively), and presence of asthma symptoms (OR 2.19). Conclusion: FEF_25–75_ deserves adequate and careful consideration in patients with asthma and normal FEV_1_ and FEV_1_/FVC, as the presence of impaired FEF_25–75_ in this subset suggests a more specific approach.

## Introduction 

Reversible bronchial obstruction is the primary physiological characteristic of asthma [1]. Measuring lung function using spirometry is the best practice for diagnosing asthma [2]. There is consensus that forced expiratory volume in one second (FEV_1_) is the most reliable marker of bronchial obstruction [3]. However, it is a common experience to measure normal FEV_1_ in the follow-up of asthma patients despite uncontrolled asthma [4]. Accordingly, the ratio between FEV_1_ and the forced vital capacity (FVC) is recognized as another marker of bronchial obstruction, but may be within the normal range in most asthma patients, as is FEV_1_ [3]. In contrast, it has been proposed that the forced vital capacity (FEF_25–75_) could be a reliable marker for revealing bronchial airflow impairment when FEV_1_ and FEV_1_/FVC are within the normal range [5]. Indeed, from 1972 to the present day, more and more evidence has accumulated regarding the reliability of FEF_25–75_ [6]. On this basis, the threshold for an impaired FEF_25–75_ was defined as < 65% of the predicted value, as reported in previous studies [5]. 

Impaired FEF_25–75_ can reflect airway hyperresponsiveness, inflammation, and disease severity more precisely than FEV_1_ in asthmatic adults and children [7, 8, 9, 10, 11]. Moreover, decreased FEF_25–75_ is associated with bronchial reversibility and response to drugs and biologics [12, 13, 14]. In addition, in the presence of normal FEV_1_ and FEV_1_/FVC, impaired FEF_25–75_ may predict possible asthma development in patients with allergic rhinitis or poor control in patients with asthma [15, 16]. Accordingly, patients with mild asthma severity frequently have normal FEV_1_ and FEV_1_/FVC. As a result, FEF_25–75_ could be a fruitful spirometric parameter in this subset of patients. 

With this background, the present study investigated the role of FEF_25–75_ in outpatients with asthma seen in daily practice. The aim was to identify feasible parameters associated with an impaired (or decreased) FEF_25–75_ value (such as < 65% of the predicted value) and to show how useful it can be in clinical practice to find an impaired FEF_25–75_ value. 

## Materials and methods 

This cross-sectional study included outpatients who consecutively visited an allergy clinic for a routine assessment. The inclusion criterion was diagnosis of asthma based on GINA criteria [1]. The exclusion criteria were: lung disease other than asthma and acute or chronic lower respiratory infections. 

The visit included history, clinical examination, lung function testing, self-administration of the Asthma Control Test (ACT) questionnaire, and asthma control assessment according to the GINA criteria [1]. 

Asthma symptoms included dyspnea, chest tightness, wheezing, and cough. Rhinitis symptoms included nasal itching, sneezing, watery rhinorrhea, and nasal congestion. Allergic rhinitis was diagnosed based on the consistency between sensitization and history [12, 15]. 

The lung function was tested with a computer-assisted spirometer (Pulmolab 435-spiro 235, Morgan, Haverhill, MA, USA; predictive values ECCS 1993). This spirometer fulfilled the American Thoracic Society/European Respiratory Society standards according to guidelines, and spirometry was performed as stated by the ERS criteria, with the execution of four measurements, choosing the best one [3, 17]. 

In this study, impaired spirometric variables were defined as: < 65% of predicted for FEF_25–75,_ < 80% of predicted for FEV_1_ and FVC, and < 0.70 for FEV_1_/FVC, as suggested in the GINA document [1]. 

Data used in this study were obtained as part of routine medical care. The Internal Review Board (IRB) of the Casa di Cura Villa Montallegro**, **Genova, Italy, approved the medical procedures performed during the routine clinical visits, including spirometry. Written informed consent was signed by participants as routinely required by local IRB. 

As appropriate, descriptive analysis of baseline characteristics was reported as count, percentage, and mean with standard deviation. Univariate and subsequent backward logistic regression analysis (variables with p < 0.10 entered into the model) were performed to detect factors significantly associated with impaired FEF_25–75_. The significance level (an error) was set at 0.05. SPSS v.24 was used for computation. 

## Results 

This study included 439 subjects (181 females and 255 males; mean age 39 ± 16.53 years). Table 1 summarizes the primary demographic and clinical outcomes in the whole sample. Then, patients were stratified into two groups considering FEF_25–75_: normal or impaired (< 65% of predicted). At univariate analysis, patients with impaired FEF_25–75_ had more frequently impaired FEV_1_ and FEV_1_/FVC (OR 0.06, p < 0.001, and OR 0.03, p < 0.001, respectively), asthma symptoms, asthma signs at examination, inadequate asthma control, and low ACT (OR 2.53, p < 0.001; OR 3.79, p < 0.001; OR 0.44, p < 0.001; and OR 0.94, p = 0.01, respectively) than subjects with normal FEF_25–75._

Also, at multivariate analysis, patients were stratified considering FEV_1_ or FVC outcomes. Consequently, the multivariate analyses showed that patients with impaired FEF_25–75_ were older (OR 1.04, p < 0.001 or OR 1.03, p = 0.006, respectively) and had lower FVC (OR 0.95, p < 001), FEV_1_ (OR 0.91, p < 0.001), and FEV_1_/FVC (OR 0.84, p < 0.001, or 0.79, p < 0.001, respectively). 

Next, to obtain a more convincing outcome, we excluded patients with impaired FEV_1_ (< 80%) and FEV_1_/FVC (<0.70) and subdivided them based on FEF_25–75_ values (Table 2). Patients with impaired FEF_25–75_ were older (OR 1.04, p = 0.001), had lower FVC, FEV_1_, and FEV_1_/FVC (OR 0.96, p < 0.001; OR 0.91, p < 0.001; OR 0.85, p < 0.001, respectively), and had more frequent asthma symptoms (OR 2.19, p = 0.026) than subjects with normal FEF_25–75._

## Discussion 

This cross-sectional study aimed to investigate the role of FEF_25–75_ in patients with asthma evaluated in a clinical setting. The findings showed that FEF_25–75_ was impaired in 31% of the outpatients. Moreover, considering only subjects with normal FEV_1_ and FEV_1_/FVC, such as those without bronchial obstruction, FEF_25–75_ was impaired in 19.6% subjects. In this subset, impaired FEF_25–75_ was associated with low FEV_1_ and FEV_1_/FVC values (OR 0.91 and 0.85, respectively) and presence of asthma symptoms (OR 2.19). Thus, the presence of impaired FEF_25–75_ in unobstructed asthmatic subjects might suggest a more stringent approach. 

Spirometry is an essential tool for diagnosing asthma and correctly managing patients with asthma. Undoubtedly, FEV_1_ and FEV_1_/FVC are reliable markers of bronchial obstruction. However, there is growing interest in the diagnostic and prognostic role of FEF_25–75_ in practical asthma management [18, 19, 20]. 

The current study investigated the practical significance of FEF_25–75_ in a cohort of adult outpatients with asthma routinely seen as outpatients. 

Interestingly, only 11.4% of patients presented bronchial obstruction, documented by consistently impaired FEV_1_ and FEV_1_/FVC. Moreover, asthma was well-controlled (GINA criteria) only in 50.2% of subjects. This finding underscores the relevance of this issue and requires adequate attention in clinical practice. 

Stratifying all asthmatic subjects by FEF_25–75_ values, patients with impaired FEF_25–75_ showed an overall clinical picture worse than those with normal FEF_25–75_. These findings were confirmed when patients with overt bronchial obstruction were excluded from the calculation. Namely, patients with impaired FEF_25–75_ but normal FEV_1_ predicted values and FEV_1_/FVC had lower FVC, FEV_1_, and FEV_1_/FVC values than patients with these parameters within the normal range. In addition, patients with impaired FEF_25–75_ more frequently reported asthma symptoms in the past month. Accordingly, only 38.6% of patients with impaired FEF_25–75_ had controlled asthma compared with 58.4% of those with normal FEF_25–75_. 

As a result, subjects with impaired FEF_25–75_ deserve significant attention, despite normal values of the customary parameters, such as FVC, FEV_1_, and FEV_1_/FVC. It is noteworthy that many physicians only assess these parameters when performing or evaluating spirometry in asthmatic patients. 

These data confirm the clinical relevance of FEF_25–75_ assessment as demonstrated in a series of studies in asymptomatic subjects, asthmatics, and patients with allergic rhinitis [5]. Indeed, a review reported that impaired FEF_25–75_ might precede FEV_1_ impairment and is associated with bronchial hyperreactivity, positive response to bronchodilation testing, and increased fractional exhaled nitric oxide (FeNO) [5]. Consequently, it has been envisaged that impaired FEF_25–75_ might be a surrogate functional marker of early bronchial involvement in allergic rhinitis, predicting subsequent asthma development [21]. So, it has been concluded that impaired FEF_25–75_ might be considered an early marker of bronchial obstruction. In addition, impaired FEF_25–75_ is associated with worse asthma progression, as documented by the high prevalence of inadequate asthma control. 

Contrarily, FEF_25–75_ is one of the first parameters to improve after successful treatment [14]. Therefore, this study supports the clinical usefulness of determining FEF_25–75_ in asthma patients, as recently underscored [22]. In addition, it has been recently demonstrated that reduced FEF_25–75_ was predictive for positive methacholine challenge test in subjects with normal FEV_1_ and FEV_1_/FVC values. In other words, an impaired FEF_25–75_ value (< 65% of predicted) suggested the presence of bronchial hyperreactivity [23]. 

However, the present study has limitations, including the cross-sectional design and data collection at only one center, the lack of measurement of inflammatory biomarkers, mainly concerning the FeNO assessment, and the analysis of only limited clinical data. 

As asthma is a condition defined by variability in lung function and symptoms, longitudinal data would significantly strengthen the study design. In addition, it should be noted that the determination of FEF_25–75_, unlike FEV_1_, depends on the determination of FVC. This could be a disadvantage. Moreover, the main limitation of this study was the lack of calculating spirometry parameters using the Global Lung Function Initiative reference values [3]. This limitation means that the results obtained must be viewed with appropriate caution. Moreover, it also has to be noted that in some studies there has been some skepticism regarding the interpretation of FEF_25–75_ [24, 25]. On the other hand, it has to be underlined that this study concerned a subset of asthmatic patients with mild asthma, as only 11.4% had bronchial obstruction. As a result, impaired FEF_25–75_ might be a fruitful diagnostic and prognostic parameter in patients with mild asthma. 

In conclusion, FEF_25–75_ deserves adequate and careful consideration in patients with asthma and normal FEV_1_ and FEV_1_/FVC, as the presence of impaired FEF_25–75_ in this subset suggests a more careful management. 

## Funding 

No funding. 

## Conflict of interest 

The authors have no conflict of interest to declare. 

**Table 1.** Demographic, clinical, and functional characteristics in 439 patients with asthma*.

		Total (N = 439)	Impaired: FEF_25–75 _ (N = 136; 31%)	Normal FEF_25–75 _ (N = 303; 69%)	Univariate OR (95% CI; p value)	Multivariate (with FEV_1_) OR (95% CI; p value)	Multivariate (with FVC) OR (95% CI; p value)
FEF_25–75_ (% predicted)		77.9 ± 25.76	49.0 ± 11.40	90.8 ± 19.05			
Age		39.0 ± 16.53	45.5 ± 15.59	36.0 ± 16.11	1.04 (1.02 – 1.05); < 0.001	1.04 (1.02 – 1.06); < 0.001	1.03 (1.01 – 1.04); 0.006
Sex	Females	255 (58.5%)	76 (56.7%)	179 (59.3%)	0.69		
	Males	181 (41.5%)	58 (43.3%)	123 (40.7%)			
Rhinitis diagnosis		381 (86.8%)	114 (83.8%)	267 (88.1%)	0.28		
FVC (% predicted)		102.9 ± 16.11	97.7 ± 17.76	105.2 ± 14.77	0.97 (0.96 – 0.98); < 0.001		0.95 (0.93 – 0.97); < 0.001
FEV_1_ (% predicted)		96.5 ± 15.75	84.4 ± 15.96	101.8 ± 12.33	0.90 (0.88- 0.92); < 0.001	0.91 (0.89 – 0.94); < 0.001	
FEV_1_ < 80%		50 (11.4%)	42 (30.9%)	8 (2.6%)	0.06 (0.03 – 0.13); < 0.001		
FEV_1_/FVC		79 ± 0.09	70.2 ± 0.09	0.83 ± 0.07	0.82 (0.78 – 0.85); < 0.001	0.84 (0.80 – 0.89); < 0.001	0.79 (0.75 – 0.84); < 0.001
FEV_1_/FVC < 0.70		50 (11.4%)	45 (33.1%)	5 (1.7%)	0.03 (0.01 – 0.09) < 0.001		
Asthma symptoms		281 (64.2%)	105 (77.8%)	176 (58.1%)	2.53 (1.59 – 4.02); < 0.001	2.18 (1.16 – 4.11); 0.016	2.09 (1.14 – 3.86); 0.017
Rhinitis symptoms		233 (53.4%)	77 (57.5%)	156 (51.7%)	0.31		
Asthma signs at examination		37 (8.6%)	22 (16.7%)	15 (5.0%)	3.79 (1.9 – 7.57) < 0.001		
Asthma control (GINA criteria)	Uncontrolled	54 (12.5%)	31 (23.1%)	23 (7.7%)	0.44 (0.32 – 0.59); < 0.001		
	Poorly controlled	161 (37.3%)	59 (44.0%)	102 (34.2%)			
	Controlled	217 (50.2%)	44 (32.8%)	173 (58.1%)			
ACT		20.0 ± 4.40	19.2 ± 4.83	20.4 ± 4.14	0.94 (0.90 – 0.99); 0.010		

*Asthma diagnosis was based on GINA criteria. FEF_25–75_ = forced expiratory flow between 25 and 75% of vital capacity. FVC = forced vital capacity. FEV_1_ = forced expiratory volume in one second. ACT = asthma control test. Data are expressed as mean + standard deviation.

**Table 2.** Demographic, clinical, and functional characteristics in 362 patients with asthma and with normal FEV1 and FEV1/FVC values.

		Total (N = 362)	Impaired FEF_25–75 _ (N = 71; 19.6%)	Normal FEF_25–75 _ (N = 291; 80.4%)	Univariate p-value	Multivariate (with FEV_1_) OR (95% CI; p-value)	Multivariate (with FVC) OR (95% CI; p-value)
FEF_25–75_ (% of predicted)		83.7 ± 22.89	54.2 ± 8.60	90.9 ± 9.19			
Age		38.2 ± 16.44	45.8 ± 15.32	36.3 ± 16.19	< 0.001	1.04 (1.02 – 1.06); 0.001	1.02 (1.00 – 1.04); 0.018
Sex	Females	222 (61.3%)	49 (69.0%)	173 (59.5%)	0.18		
	Males	140 (38.7%)	22 (31.0%)	118 (40.5%)			
Rhinitis diagnosis		316 (87.3%)	61 (85.9%)	255 (87.6%)	0.85		
FVC (% of predicted)		105.1 ± 14.34	102.5 ± 15.32	105.7 ± 14.05	0.024		0.96 (0.93 – 0.98); < 0.001
FEV_1_ (% of predicted)		100.7 ± 12.15	92.5 ± 10.70	102.7 ± 11.65	< 0.001	0.91 (0.88 – 0.94); < 0.001	
FEV_1_/FVC		0.82 ± 0.07	0.77 ± 0.06	0.83 ± 0.07	< 0.001	0.85 (0.80 – 0.90); < 0.001	0.81 (0.76 – 0.86); < 0.001
Asthma symptoms		216 (59.8%)	49 (70.0%)	167 (57.4%)	0.07	2.19 (1.10 – 4.36); 0.026	2.32 (1.21 – 4.42); 0.011
Rhinitis symptoms		183 (51.0%)	37 (53.6%)	146 (50.3%)	0.72		
Asthma signs at examination		18 (5.1%)	4 (5.9%)	14 (4.9%)	0.97		
Asthma control (GINA criteria)	Uncontrolled	28 (7.9%)	8 (11.4%)	20 (7.0%)	0.011		
	Poorly controlled	134 (37.6%)	35 (50.0%)	99 (34.6%)			
	Controlled	194 (54.5%)	27 (38.6%)	167 (58.4%)			
ACT		20.4 ± 4.19	20.0 ± 4.56	20.5 ± 4.10	0.51		

FEF_25–75_ = forced expiratory flow between 25 and 75% of vital capacity. FVC = forced vital capacity. FEV_1_ = forced expiratory volume in one second. ACT = asthma control test. Data are expressed as mean ± standard deviation.
